# Adaptation and validation of a German version of the Strengths Use and Deficit Correction (SUDCO) questionnaire

**DOI:** 10.1371/journal.pone.0245127

**Published:** 2021-01-07

**Authors:** Timo Lorenz, Kathrin Heinitz, Clemens Beer, Marianne van Woerkom

**Affiliations:** 1 Department of Psychology, Medical School Berlin, Berlin, Germany; 2 Department of Work and Organizational Psychology, Freie Universität Berlin, Berlin, Germany; 3 Department of Human Resource Studies, Tilburg University, Tilburg, The Netherland; Aalborg University, DENMARK

## Abstract

The Strength Use and Deficit Correction (SUDCO) Questionnaire has been shown to be a reliable instrument for the measurement of its four dimensions *perceived organizational support for strengths use*, *perceived organizational support for deficit correction*, *strengths use behavior*, and *deficit correction behavior* in the context of organizations. This paper aims to adapt and validate the SUDCO for the German-speaking population (SUDCO-G). Three studies were conducted. Confirmatory factor analyses and correlations with other psychological constructs on the data of three German samples (*N*_*1*_ = 302; *N*_*2*_ = 243, N_*3*_ = 295) were performed. The twenty-four item SUDCO-G exhibits the anticipated factorial structure with four factors and an acceptable model fit in all three studies (*CFI* = .920-.937, *TLI* = .911-.929, *RMSEA* = .063-.079, *SRMR* = 0.52-.075). The associations of the four dimensions to other constructs concur with previous findings (study 2) and the subscales of the SUDCO-G also show positive relations with general strengths use, meaning of work and Psychological Capital (study 3). We conclude that the SUDCO-G is a reliable and valid instrument for the use in the German-speaking population.

## Introduction

In the face of the positive psychology movement initiated by Csikszentmihalyi and Seligman [1] individual strengths have been in the focus of a growing body of research. Two major perspectives can be distinguished in the literature: *possessing* strengths and the actual *use* of them [2]. While possessing and knowing strengths has been positively associated with life satisfaction, a pleasurable and meaningful existence, vitality and well-being [3, 4], Govindji and Linley [5] as well as Wood et al. [2] suggested that merely knowing one’s strengths is not enough but that *using* one’s strengths is what yields the most valuable outcomes. Govindji and Linley [5] supported their claims by showing that strength use improves subjective and psychological well-being, whereas the knowledge of one’s strengths was no significant predictor for either. Strength use is associated with less stress and depressive symptoms and more engagement at work, meaning of work, positive affect, self-esteem, self-efficacy, vitality and overall well-being [2, 5–8]. Harzer and Ruch [6] further claimed that these positive outcomes of strength use are irrespective of the kind of strengths that are used.

Harzer and Ruch [6] reported that activities at the workplace that are congruent with one’s strengths are associated with higher job satisfaction, engagement and meaning at work. This is also supported by Stander, Mostert, and de Beer [9], who found a significant positive relation between strength use behavior and work engagement as well as productivity in a sample of call center operators. A study with future teachers by Çelebi, Krahé, and Spörer [10] found participants in an intervention focusing on their individual strengths to show increased self-efficacy and professional self-regulation.

Fixing one’s weaknesses on the other hand can yield positive outcomes as well: In the same study by Çelebi et al. [10] an intervention group focusing on professional weaknesses or deficits, the tasks which do not come naturally to an individual,was tested. Working on one’s deficits was found to increase self-efficacy and professional self-regulation. Furthermore, this deficit correction, the extent to which an organization actively supports individuals to correct their deficits by narrowing the gap between the actual and desirable behaviors and performance through i.e. coaching, training, feedback, or on the job learning processes, was positively related to hope, efficacy, engagement and life satisfaction and negatively related to burnout. Deficit correction behavior was furthermore associated with a better perception of the personal fit with a study course [8, 11–13].

Van Woerkom et al. [14] stated in accordance with Linley, Joseph, Harrington, and Wood [15] as well as Seligman, Parks, and Steen [16], that the ‘*ultimate challenge for positive psychology is to synthesize positive and negative aspects of human experience’* (p. 960). Focussing on strengths use and correcting deficits have both beneficial effects, therefore neglecting one side will not paint the full picture. This is also supported by the results of Çelebi et al. [10]: whereas strengths use support and deficit correction behaviors both had effects on several outcomes, the combination of strengths use and deficit correction had the highest effects on self-efficacy and self-regulation.

There are several instruments to assess a person’s strengths (CFS, [17]; VIA-IS, [18]) and the *use* of them (SUS, [5]). In a recent study, Van Woerkom et al. [14] developed and validated an instrument that focuses not only on strength use behavior (SUB) but also on deficit correction behavior (DCB), thereby providing an instrument assessing both sides: The Strengths Use and Deficit Correction (SUDCO) Questionnaire.

As the questionnaire was developed for work contexts, Van Woerkom et al. [14] did not just focus on the individual and the mere behaviors of strength use or deficit correction, but also on the role of the organization. They distinguished perceived organizational support for strengths use (POSSU) and perceived organizational support for deficit correction (POSDC). POSSU is defined as ‘*employees’ perceptions of the […] policies*, *practices*, *and procedures in their organization concerning the identification*, *development*, *use*, *and appreciation of their talents and strengths’* [19, p 4]. POSDC is referred to as ‘*employees’ beliefs concerning the extent to which the organization actively supports them to correct their deficits*’ [14, p 961].

Organizational support for strengths use can decrease absenteeism and burnout [20, 21] and is positively related to engagement, job performance and positive affect [9, 12, 19, 22]. Organizational support for deficit correction is associated with positive results as well: Organizational support addressing deficits through training opportunities was found to decrease stress and job demands [23]. A study by LaFleur and Hyten [24] also showed that the behavioral analysis of performance problems of hotel staff and the implementation of a resulting training package lead to increased performance. Thus, organizational support for both behaviors can result in positive outcomes for the individual and the organization, supporting the general notion that perceived organizational support can lead to valuable outcomes like positive mood, job satisfaction and increased performance [25].

The original SUDCO was developed for English speaking populations. Our paper aims at adapting and validating the SUDCO for the German-speaking population. Three studies were conducted. In study 1 the SUDCO was adapted for the German-speaking population and tested for its factorial structure. Studies 2 and 3 tested the external validity of the newly developed SUDCO-G. Furthermore, both studies offered the chance to retest the SUDCO-G to see if previous findings of factorial structure and model fit could be reapproved. Measurement invariance with the English version of the SUDCO was also examined [26].

### The Strengths Use and Deficit Correction (SUDCO) questionnaire

The SUDCO was developed using two South African samples (*N* = 338; *N* = 361). With 24 items, it presents a clear four-factor model (POSSU, POSDC, SUB, DCB) with acceptable fit indices (CFI = .92, TLI = .91, RMSEA = .08, SRMR = .05) regarding to Hu and Bentler [27] and very good reliability [14]. Similar psychometric properties could be presented in a later study by Els, Mostert, and Brouwers [28]: Using the SUDCO in a South African sample (*N* = 858) the proclaimed four-factor model was supported with very similar fit indices (CFI = .93, TLI = .92, RMSEA = .08, SRMR = .05).

Testing for convergent validity in a South African (*N* = 361) and a Dutch (*N* = 133) sample, Van Woerkom et al. [14] found all four dimensions of the SUDCO to be positively correlated with perceived supervisor support, perceived organizational support, vigor, dedication, proactive personality, personal initiative, and negatively with exhaustion and cynicism. Thus, all four dimensions were related to valuable organizational outcomes.

For the adaptation of a questionnaire into a different language, culture or country, many different issues must be considered. Beaton, Bombardier, Guillemin, and Ferraz [29] stated that ‘*the process of cross-cultural adaptation tries to produce equivalency between source and target based on content’* (p. 3186) and they furthermore claimed that there are several different scenarios, in which guidelines for cross-cultural adaptation should play an important role. Guillemin, Bombardier, and Beaton [30] and Beaton et al. [29] proclaimed five scenarios where some sort of cross-cultural adaptation is necessary. In the case of this paper the scenario is the adaptation of the original questionnaire into another language *and* another country (for more details [29]), which implicates that translation *and* cultural adaptation are both required [29, 30]. In the entire translation process we followed the guidelines of cross-cultural adaptation by Beaton et al. [29], Guillemin [31] and Guillemin et al. [30] and the guidelines of translation by Epstein, Santo, and Guillemin [32] and Peters and Passchier [33]. The final version of the translation was checked for cross-cultural equivalence to ensure that the ‘*translation is fully comprehensible’* [30, p 1422].

We derived the following hypotheses and expected the hypothesized correlations to be similar to the correlations found in the study from van Woerkom et al. [14] described above:

*Hypothesis 1*: The four-factor model with the 24 items of the original version will fit the data better than alternative models.

*Hypothesis 2*: All four dimensions of the SUDCO-G (POSSU, POSDC, SUB, DCB) are positively related with perceived supervisor support (2 a-d), vigor (2 e-h), dedication (2 i-l), proactive personality (2 m-p), and personal initiative (2 q-t).

*Hypothesis 3*: All four dimensions of the SUDCO-G (POSSU, POSDC, SUB, DCB) are negatively related with exhaustion (3 a-d) and cynicism (3 e-h).

*Hypothesis 4*: All four dimensions of the SUDCO-G (POSSU, POSDC, SUB, DCB) are positively related with general perceived organizational support (4 a-d) and perceived supervisor support (4 e-h).

*Hypothesis 5*: SUB is positively related to self-efficacy (5a) and positive affect (5b).

*Hypothesis 6*: POSSU and POSDC are positively related to job satisfaction (6 a,b) and positive affect (6 c,d).

Whereas the choice of validation criteria in study 2 followed the original study of Van Woerkom et al. [14], we examined the relation of the SUDCO-G subscales with further criteria in study 3. Huber, Webb and Höfer [34] recently validated the German strengths use scale, a scale that measures general strengths use in everyday life. As work is an important life domain where strengths can be used, we assume that general strengths use is correlated with SUB and that the support of strengths use at work also has a positive relation with general strengths use.

*Hypothesis 7*: General strengths use is positively related to SUB (7a) and POSSU (7b).

On a general level, strengths deployment is related to meaning in life [35]. Being able to use one’s strengths at work should also increase the perception of meaning at work, in the sense of finding it meaningful. Using your strengths at work is associated with higher levels of job satisfaction and perceptions of self-efficacy [2, 5–7]. Job satisfaction and self-efficacy in turn are related to experiencing work as meaningful [36]. General organizational support is also related to self-efficacy [37] and job satisfaction [38] and this relation should also hold for specific strengths related organizational support. We therefore assume:

*Hypothesis 8*: SUB (8 a) and POSSU (8 b) are positively related to meaning of work.

Psychological Capital (PsyCap) is an overarching concept including hope, self-efficacy, optimism and resilience [39] is associated with a variety of outcomes relevant for the organization like e.g. satisfaction, turnover intent or organizational citizenship behavior [40]. People high in PsyCap are more empowered and can draw upon more resources to pursue goals [41]. Using one’s strengths is related to higher levels of self-efficacy [5, 42]. It should also lead to the belief to find means and ways to accomplish a task (one aspect of hope), and hence to an expectancy of positive outcomes (optimism). Being able to use one’s strengths could also help bouncing back from adversities (resilience). As the four components of PsyCap are also highly correlated, we assume that strenghts use is correlated to the compound construct. In the diary study of Van Woerkom et al. [42] weekly strengths use mediated the relation of leader strengths use support and self-efficacy, indiciating that POSSU should also be positively related to PsyCap.

*Hypothesis 9*: SUB (9a) and POSSU (9b) are positively related to PsyCap.

## Study 1

The focus of study 1 was to examine the factor structure of the German translation of the SUDCO.

### Methods

#### Participants and procedure

Participants in this study were recruited by publishing the link to the online-survey in a multitude of online social media groups and social networks. The survey was administered in German. Participation was voluntary, inclusion criteria for participation was to be in at least a part-time employment relationship. No incentives were supplied. A sample size of at least *N* = 200 was aspired, to provide sufficient data points conducting confirmatory factor analyses (CFA) [43].

The sample consisted of a total of 317 participants. 15 participants were excluded from the analysis due to implausible demographic answers—one due to implausible age and 14 due to implausible tenure. The 302 remaining participants averaged 30.81 years of age (*SD* = 10.02), 52% were women (*n* = 157). All participants were employed with an average tenure of 56.88 months at their current job (*SD* = 79.15). The average time spent at work was 32.93 hours a week (*SD* = 12.94). The job sector of health, social policy and education was represented by most of the participants (25.8%), followed by commercial service, merchandise trade, marketing and tourism (18.9%) and natural sciences, geography and computer sciences (12.3%).

#### Demographics

Data regarding age and gender (‘*male*’, ‘*female*’, and ‘divers’) were collected. Participants were asked to choose their job sector according to the classification of occupations [44], name their specific job title, tenure and the average hours worked per week.

#### Scale construction

33 items were tested to create the SUDCO-G. These items contained the 24 final items of the original SUDCO, plus nine previously included items that have later been deleted by Van Woerkom et al. [14] due to ‘*their wordings being similar to other items*, *modification indices*, *and the face and content validity of the remaining items’* (p. 965). Although it was preferred to use the previously selected 24 items, the inclusion of the excluded nine items might be valuable to account for possible differences between the South African and the German sample.

All items were translated into German following the guidelines of Epstein et al. [32]. Two psychologists with knowledge of the construction of psychometric instruments and two lay people translated all items, respectively. All four translators were native German speakers with fully proficient English skills due to many years spent in English speaking countries. They then built an expert committee comparing and discussing the four translations [32]. The final version was then back-translated into English by a bilingual English and German teacher and then tested for equivalence [33, 45].

#### Data analysis

Following Van Woerkom et al. [14], we tested the following models conducting CFAs using the ‘*lavaan*’ package [46] of R statistical software [47]: (1) The proposed four-factor model (POSSU, POSDC, SUB, DCB) with the original 24 items and (2) a one factor model with 24 items, (3) a two-factor model with all items of POSSU and POSDC loading on one factor and all items of SUB and DCB loading on the other factor with the 24 original items, and (4) a two-factor model with all items of POSSU and SUB loading on one factor and all items of POSDC and DCB loading on the other factor with the 24 original items, and (5) the proposed four-factor model (POSSU, POSDC, SUB, DCB) with all 33 items. The fit of the structural equation model was examined using the criteria proposed by Hu and Bentler [27]. The Satorra-Bentler adjusted χ² was calculated to adjust for non-normal distributions of the variables [48]. All other analyses were performed using SPSS [49] and R statistical software [47]. Used R packages for descriptive statistics, bivariate correlations and other analyses were ‘*Hmisc*’ [50], ‘*psych*’ [51], ‘*foreign*’ [47], ‘*gdata*’ [52], ‘*HSAUR2*’ [53], ‘*GPArotation*’ [54], ‘*car*’ [55] and ‘*MVA*’ [56]. There was no missing data due to forced choice in the standardized questionnaire. To compute point biserial correlations manifest mean scores of the scales were used.

### Ethics statement

This study is in accordance with the APA ethical principles regarding research with human participants. This study does not involve any conflict of ethics, since no clinical intervention was performed. Neither were blood or tissue samples taken for study purposes.

Participants were informed before participating that their responses would be treated confidentially and anonymously and that all data would be analyzed in a generalized manner so that no conclusions could be drawn about individual persons. The participants were informed that they would give their consent by proceeding past the welcome page of the online survey. This procedure is in accordance with the Medical School Berlin ethics committee’s guidelines. There was no contact between researchers and participants. Participation in this study was voluntary. This study was approved by the ethics committee of the Medical School Berlin (MSB2020-23).

### Results

All fit indices for the five models can be found in Table 1. To evaluate the model fit the comparative fit index (CFI), the Tucker–Lewis Index (TLI), the root mean square error of approximation (RMSEA), and the standardized root mean square residual (SRMR) were used. Hu and Bentler [27] deemed it difficult to define fixed cutoff criteria for the fit indices claiming that they do not ‘*work equally well with various conditions’* (p. 27). Nonetheless they recommended cutoff levels close to .95 or higher for CFI and TLI, cut off levels close to .08 or lower for SRMR and cutoff levels close to .06 or lower for RMSEA to be acceptable [27].

**Table 1:** Measurement models for Study 1 using MLM estimator.

Model	*χ²*	*df*	*p*	CFI	TLI	RMSEA	RMSEA-90%CI	SRMR
1: Four-factor model (POSSU, POSDC, SUB, DCB) with original 24 items	541.2	246	< .001	.937	.929	.063	.056 - .070	.052
2: One-factor model with 24 items	2069.4	252	< .001	.559	.517	.155	.149 - .160	.179
3: Two-factor model (POS and behavior) with original 24 items	1817.8	251	< .001	.665	.632	.144	.138 - .150	.164
4: Two-factor model (strength use and deficit correction) with original 24 items	1250.6	251	< .001	.786	.765	.115	.109 - .120	.098
5: Four-factor model (POSSU, POSDC, SUB, DCB) with 33 items	1096.2	489	< .001	.911	.904	.064	.060 - .069	.062

Notes. POSSU = perceived organizational support for strengths use, POSDC = perceived organizational support for deficit correction, SUB = strengths use behavior, DCB = deficit correction behavior; N = 302.

The two four-factor models (models 1 and 5) present acceptable fit indices. The one-factor model (model 2) and the two two-factor models (models 3 and 4) present unacceptable fit indices and received no further consideration. Model 1 showed a significantly better fit to the data than model 5 (Δ *χ²* = 554.44, Δ *df* = 243, *p* < .001). Thus, model 5 equally received no further consideration.

For model 1 the items that had already been used by Van Woerkom et al. [14] for the original SUDCO were used for each facet (POSSU, POSDC, SUB and DCB). The result was a four-factor model with 24 items, seven items for POSSU, five items for POSDC and six items for SUB and DCB, respectively. The result of the model fit was very similar to the original study [14] and deemed acceptable due to Hu and Bentler [27], thus supporting hypothesis 1. The main factor loadings for model 1 (Table 2) presented values between .61 and .83, all of them significant on their respective factor at *p <* .01. Table 3 presents descriptive statistics, bivariate correlations and Cronbach’s *α* and McDonald’s *ω* for the study variables. Cronbach’s *α* and McDonald’s *ω* for the four scales.

**Table 2:** Factor loadings of all items of the SUDCO-G for Study 1.

Item no.	Item wording	*M*	*SD*	*factor loadings (error)*
	***POSSU***			
1.	Mein Unternehmen gibt mir die Möglichkeit, das zu tun, worin ich gut bin. (POSSU-1)	5.17	1.34	.85 (.28)
2.	Mein Unternehmen ermöglicht es mir, meine Talente einzusetzen. (POSSU-2)	4.95	1.46	.87 (.25)
3.	Mein Unternehmen stellt sicher, dass meine Stärken mit meinen Arbeitsaufgaben abgestimmt sind. (POSSU-3)	4.47	1.52	.87 (.24)
4.	Mein Unternehmen holt das Meiste aus meinen Talenten raus. (POSSU-4)	4.05	1.60	.89 (.22)
5.	Mein Unternehmen ist fokussiert auf das, worin ich gut bin. (POSSU-5)	4.28	1.64	.84 (.29)
6.	Mein Unternehmen setzt meine Stärken ein. (POSSU-6)	4.68	1.54	.87 (.24)
7.	Mein Unternehmen erlaubt es mir meine Arbeit so auszuüben, wie es am besten zu meinen Stärken passt. (POSSU-7)	4.65	1.60	.83 (.31)
	***POSDC***			
8.	In meinem Unternehmen erhalte ich Trainings, um meine Schwächen zu verbessern. (POSDC-1)	3.15	1.88	.73 (47)
9.	Mein Unternehmen verlangt von mir, an meinen Defiziten zu arbeiten. (POSDC-3)	3.91	1.78	.79 (.38)
10.	Mein Entwicklungsplan im Unternehmen zielt darauf ab, meine Schwächen zu verbessern. (POSDC-4)	3.29	1.80	.87 (.25)
11.	In meinem Unternehmen greifen Leistungsbeurteilungen meine Entwicklungspotenziale auf. (POSDC-5)	3.27	1.94	.74 (.45)
12.	Mein Unternehmen erwartet von mir, dass ich mich in den Dingen verbessere, in denen ich nicht gut bin. (POSDC-7)	3.90	1.77	.70 (.51)
	***SUB***			
13.	In meinem Beruf mache ich das Beste aus meinen Stärken. (SUB-1)	5.32	1.29	.85 (.27)
14.	Ich organisiere meine Arbeit so, dass sie zu meinen Stärken passt. (SUB-2)	5.32	1.30	.86 (.26)
15.	Bei meiner Arbeit profitiere ich von meinen Stärken. (SUB-3)	5.44	1.35	.89 (.21)
16.	Ich suche Möglichkeiten um meine Arbeit in einer Art und Weise auszuführen, die am besten zu meinen Stärken passt. (SUB-4)	5.47	1.28	.79 (.37)
17.	In meinem Beruf versuche ich meine Talente so oft wie möglich einzusetzen. (SUB-6)	5.41	1.35	.74 (.45)
18.	Ich nutze meine Stärken bei meiner Arbeit. (SUB-8)	5.45	1.22	.80 (.36)
	***DCB***			
19.	Ich übe Aktivitäten aus, um meine Schwachstellen auf Arbeit weiterzuentwickeln. (DCB-2)	3.97	1.42	.70 (.51)
20.	In meinem Beruf bemühe ich mich, meine Defizite zu verbessern. (DCB-4)	4.50	1.40	.79 (.37)
21.	Auf der Arbeit bitte ich um Feedback bezüglich der Bereiche, in denen ich mich noch entwickeln kann. (DCB-5)	3.87	1.80	.67 (.55)
22.	Auf der Arbeit suche ich nach Trainingsmöglichkeiten, um meine Schwächen zu verbessern. (DCB-6)	3.91	1.61	.77 (.40)
23.	In meinem Beruf arbeite ich an meinen Defiziten. (DCB-7)	4.06	1.49	.88 (.23)
24.	Ich denke darüber nach, wie ich Dinge in meinem Beruf verbessern kann, in denen ich nicht gut bin. (DCB-8)	4.61	1.41	.73 (.47)

Notes. POSSU = perceived organizational support for strengths use, POSDC = perceived organizational support for deficit correction, SUB = strengths use behavior, DCB = deficit correction behavior; factors: 1 = POSSU, 2 = POSDC, 3 = SUB, 4 = DCB; factor loadings are not reported when < .1; N = 302.

**Table 3:** Descriptive statistics and inter-correlations for Study 1.

Variables	*M*	*SD*	*α*	*ω*	1.	2.	3.	4.	5.	6.	7.
1. POSSU	4.61	1.35	.95	.97	1						
2. POSDC	3.51	1.50	.87	.91	.37***	1					
3. SUB	5.40	1.11	.93	.95	.65***	.29***	1				
4. DCB	4.15	1.22	.89	.92	.25***	.51***	.28***	1			
5. Age	30.81	10.02	-	-	.03	-.13*	.18**	-.15**	1		
6. Tenure	56.99	79.15	-	-	-.03	-.07	.19***	-.12*	.72***	1	
7. Hours	32.93	12.94	-	-	.14*	.28***	.11	.18**	.18**	.21***	1

Notes. POSSU = perceived organizational support for strengths use, POSDC = perceived organizational support for deficit correction, SUB = strengths use behavior, DCB = deficit correction behavior, Age = age in years, Tenure = tenure in months, Hours = hours worked per week; p-scores:

* < .05,

** < .01,

*** < .001; N = 302.

### Conclusion

The final choice to continue the process of the adaptation and validation of the SUDCO-G in study 2 was model 1. Model 1 showed a significantly better fit than model 5 and provides a theoretical basis for the selection of the 24 used items. Model 1 also allows for better comparability to the original version of the SUDCO, as the exact same items are used. Thus model 1 was the preferential choice in this case. We therefore proceeded with the validation of the SUDCO-G in study 2 with the 24 items used in model 1.

## Study 2

The focus of study 2 was to re-examine the factor structure of 24 the item version of the German translation of the SUDCO as well as the examination of its relationships with criteria that were also examined by Van Woerkom et al. [14; _see Hypotheses 2–6_].

### Methods

#### Participants and procedure

Participants of study 2 were recruited analogous to study 1. The sample consisted of a total of 255 participants. 12 participants were excluded from the analysis due to implausible answers—two due to implausible tenure and ten due to implausible answers on the third item of job satisfaction, where they had to rate the percentage of time they feel satisfied, unsatisfied or neutral with their job in general. For the ten excluded participants, the percentages did not add up to 100 percent. The remaining 243 participants were on average 33.19 years of age (*SD* = 10.84), 60.9% were women (*n* = 148), 38.7% were men (*n* = 94), whereas one participant declared the sex ‘other’. Participants were employed with an average tenure of 63.29 months at their current job (*SD* = 87.33). The average time spent at work was 33.81 hours a week (*SD* = 11.46). The job sector of health, social policy and education was represented by the most participants (49.4%), followed by natural sciences, geography and computer sciences (12.8%) and the military (9.5%).

### Measures

The four dimensions of the SUDCO were measured with the instrument that was validated in study 1. Participants rated the 24 items of the SUDCO-G on a 7-point scale from 1 = *‘almost never’* to 7 = *‘almost always’* (e.g. ‘*I use my strengths at work*.’).

***Vigor and dedication*** were measured using the respective subscales of the Utrecht Work Engagement Scale (UWES-9 [57]). Using a 7-point scale from 1 = *‘never’* to 7 = *‘always’* participants rated three items, respectively (e.g. ‘*At work*, *I feel bursting with energy*.’).

***Proactive personality*** was measured with the Proactive Attitude Scale [58]. On a 4-point scale from 1 = *‘not at all true’* to 4 = *‘exactly true’* participants rated eight given statements (e.g. ‘*I can choose my own actions*.’).

***Personal initiative*** was measured with a questionnaire developed by Frese, Fay, Hilburger, Leng, and Tag [59]. Using a 5-point scale from 1 = *‘not at all true’* to 5 = *‘very true’* participants rated seven given items (e.g. ‘*I actively attack problems*.’).

#### Exhaustion and cynicism

Exhaustion was measured using the respective subscale of the Maslach Burnout Inventory [60]. Participants used a 7-point scale from 1 = *‘never’* to 7 = *‘always’* to rate the nine items (e.g. ‘*I feel burned out because of my work*.’). Cynicism was measured using the cognitive subscale of a questionnaire developed by Abhari and Schilling [61]. Participants used a 6-point scale from 1 = *‘absolutely not true’* to 6 = *‘absolutely true’* to rate the six items (e.g. ‘*What is said in my organization and what is actually done*, *are two different things*.’).

#### Perceived organizational support and perceived supervisor support

Both constructs were measured using three translated high loading items of the Survey of Perceived Organizational Support (SPOS [62]). The item selection followed the recommendations of Eisenberger, Stinglhamber, Vandenberghe, Sucharski, and Rhoades [63]. One of the recommended items was substituted due to a potential overlap with strengths use (‘*The organization is willing to extend itself in order to help me perform my job to the best of my ability’* was replaced by ‘*The organization cares about my general satisfaction at work’* and adapted for a supervisor perspective as suggested in [63]). Rhoades and Eisenberger [25] claim the use of a shorter version of the SPOS to not be problematic, as it is unidimensional and has high internal reliability. Items were rated on a 7-point scale from 1 = *‘strongly disagree’* to 5 = *‘strongly agree’* (e.g. ‘*My organization strongly considers my goals and values*.’).

#### Positive affect

The Positive and Negative Affect Schedule (PANAS [64]) was used to measure positive affect and negative affect. Using a 5-point scale from 1 = *‘very slightly or not at all’* to 5 = *‘very much’* participants responded with twenty items to the question asking how they felt ‘*during the past two weeks*’. Only the items for positive affect were used to measure positive affect.

#### Self-efficacy

The German Generalized Self-Efficacy Scale (GSE [65]) was used to measure self-efficacy. Ten items were rated on a 6-point scale from 1 = *‘not at all true’* to 7 = *‘completely true’* (e.g. ‘*If I am in trouble*, *I can usually think of a solution*.’).

#### Job satisfaction

Three items were used to measure job satisfaction [66, 67]. The first item measures general job satisfaction (‘*All things considered are you satisfied with your job*?’), which participants could answer with ‘*yes*’ or ‘*no*’. The second item (‘*How satisfied are you with your job in general*?’) was rated using a 5-point scale from 1 = *‘very dissatisfied’* to 5 = *‘very satisfied’*. The third item asked participants to rate the percentage of time they feel satisfied, unsatisfied or neutral with their job in general (e.g. ‘*The percent of time I feel satisfied with my present job*.’). The analysis was conducted using the mean-score of the z-standardized items.

#### Data analysis

The data analysis was performed analogous to study 1 using R statistical software [47] and SPSS [49].

### Results

Results of the CFA for the SUDCO-G showed an acceptable fit according to Hu and Bentler [27]: *χ²* (246) = 606.2, *p <* .001, CFI = .920, TLI = .911, RMSEA [95% CI] = .078 [.017 - .085], SRMR = .075. The result was very similar to the model fit in the original study [14] and to the model fit in study 1, therefore supporting hypothesis 1 and reapproving the results of study 1. The factor loadings for model 1 presented values between .59 and .91, all of them significant on their respective factor at *p <* .01.

Table 4 presents descriptive statistics, bivariate correlations and Cronbach’s *α* and McDonald’s *ω* for the study variables. Cronbach’s *α* and McDonald’s *ω* for the four scales of the SUDCO-G were very good. The reliability for all other scales were good to very good, except for the scale to assess proactive personality (*α* = .62, *ω* = .72). Due to the very high correlation between POSSU and perceived organizational support (*r* = .73) and POSSU and perceived supervisor support (*r* = .65) we investigated if the constructs are distinct from each other. The CFAs showed, that a two-factor model with POSSU and perceived organizational support did fit the data significantly better than a one factor-model with both constructs loading on the same factor (Δ *χ²* = 51.12, Δ *df* = 1, *p* < .001). The same was found for POSSU and perceived supervisor support (Δ *χ²* = 161.97, Δ *df* = 1, *p* < .001), thus supporting in both cases the distinction of the constructs from each other.

**Table 4:** Descriptive statistics and inter-correlations for Study 2 (N = 243).

Variables	*M*	*SD*	*α*	*ω*	1.	2.	3.	4.
1. POSSU	4.24	1.50	.96	.97	1			
2. POSDC	3.20	1.66	.90	.94	.36***	1		
3. SUB	5.43	1.10	.93	.96	.59***	.12	1	
4. DCB	4.55	1.29	.90	.93	.25***	.44***	.39***	1
5. Age	33.19	10.84	-	-	.14*	-.23 ***	.35***	.13*
6. Tenure	63.29	87.33	-	-	.18**	-.08	.29***	.13*
7. Hours	33.81	11.46	-	-	.06	.23***	.15*	.21**
8. Vig	4.67	1.21	.82	.84	.50***	.21***	.48***	.38***
9. Ded	4.99	1.33	.89	.89	.58***	.31***	.56***	.35***
10. ProP	3.08	0.37	.62	.72	.16*	.23***	.21**	.29***
11. PersI	3.89	0.54	.78	.84	.16*	.18**	.33***	.34***
12. Exh	2.48	0.88	.88	.92	-.34***	-.02	-.29***	-.09
13. Cyn	3.22	1.14	.93	.96	-.54***	-.23***	-.23***	-.09
14. PSS	4.47	1.47	.89	.88	.65***	.42***	.33***	.32***
15. POS	4.30	1.33	.88	.90	.73***	.37***	.35***	.20***
16. PA	3.23	0.74	.90	.92	.45***	.22***	.40***	.36***
17. NA	1.72	0.59	.83	.88	-.33***	.00	-.31***	-.04
18. SE	4.32	0.61	.88	.91	.15*	.10	.24***	.12
19. JS ^a^	0	0.84	.81	.94	.64***	.26***	.46***	.19**

Notes. POSSU = perceived organizational support for strengths use, POSDC = perceived organizational support for deficit correction, SUB = strengths use behavior, DCB = deficit correction behavior, Age = age in years, Tenure = tenure in months, Hours = hours worked per week, Vig = vigor, Ded = dedication, ProP = proactive personality, PersI = personal initiative, Exh = exhaustion, Cyn = cynicism, PSS = perceived supervisor support, POS = perceived organizational support, PA = positive affect, NA = negative affect, SE = self-efficacy, JS ^a^ = job satisfaction; p-scores:

* < .05,

** < .01,

*** < .001.

^a^ standardized z-scores.

POSSU showed positive correlations with vigor, dedication and perceived supervisor support, and small positive correlations with proactive personality and personal initiative. POSDC was found to have moderate positive correlations with dedication and perceived supervisor support, and small positive correlations with vigor, proactive personality and personal initiative. SUB presented a high positive correlation with dedication, moderate positive correlations with vigor, personal initiative and perceived supervisor support and a small positive correlation with proactive personality. DCB showed moderate positive correlations in approximately the same range with all five constructs. All correlations are interpreted using the guidelines by Cohen [68]. They fully support hypothesis 2 (a-t).

As expected, both POSSU and SUB showed significant negative correlations with exhaustion and cynicism. POSDC was found to have a significant negative correlation with cynicism. POSDC and exhaustion presented no significant correlation. DCB showed no significant correlations with either exhaustion or cynicism, although both correlations leaned towards the expected direction. Thus, hypotheses 3 a, c, e, f and g are supported, whereas 3 b, d and h are not supported.

Hypothesis 4 (a-h) are fully supported as all four dimensions of the SUDCO-G presented the expected significant positive correlations with general perceived organizational support and perceived supervisor support. POSSU showed the highest correlation with both support scales. Hypotheses 5 a and b are equally supported. SUB shows significant positive correlations with positive affect and self-efficacy, as expected. POSSU and POSDC show significant positive correlations with positive affect and job satisfaction, therefore supporting hypotheses 6 a through d as well.

### Conclusion

Study 2 supported the findings from study 1, showing that the SUDCO-G can be adapted for the German-speaking population with the same items, factor structure and similar acceptable model fit indices to the original questionnaire. With nearly all correlations being in a similar range to previous findings by Van Woerkom et al. [14], the results testing for external validity were largely as expected. Thus, the SUDCO-G appears to be a reliable and valid instrument to measure the four dimensions POSSU, POSDC, SUB and DCB in the German-speaking population.

## Study 3

The focus of study 3 was again to re-examine the factor structure of the 24 item version of the German translation of the SUDCO as well as the examination of its relationships with general strengths use, as well as meaning of work and PsyCap.

### Methods

#### Participants and procedure

Participants of study 3 were recruited analogously to study 1 and 2. The sample consisted of a total of 300 participants. 5 participants were excluded from the analysis due to non-employment status. The remaining 295 participants averaged 31.24 years of age (*SD* = 10.65), 66% were women (*n* = 196), 33% were men (*n* = 96), three participants declared the sex ‘other’. The participants were employed with an average tenure of 51.12 months at their current job (*SD* = 87.33). The average time spent at work was 30.47 hours a week (*SD* = 15.15). The job sector of health, social policy and education was represented by the most participants (25%), followed by commercial services, merchandise trade, marketing and tourism (22%), and liberal arts, media, business sciences, arts, culture and design (19%).

### Measures

#### POSSU, POSDC, SUB and DCB

The SUDCO-G was used as in study 2.

#### General strengths use

General strengths use was measured using the German version of the strengths use scale [34]. The scale measures a single factor with 14 items using a 7-point scale from 1 = ‘*strongly disagree*’ to 7 = ‘*strongly agree*’ (e.g. ‘*I am able to use my strengths in lots of different ways*.’).

#### Meaning of work

The Work and Meaning Inventory [69] was used to measure meaning of work. Using a 5-point response format ranging from 1 = ‘*absolutely untrue*’ to 5 = ‘*absolutely true*’ participants rated to what extent the ten given statements (e.g. ‘*My work helps me make sense of the world around me*.*’*) applied to them.

#### Psychological Capital (PsyCap)

PsyCap was measured with the Compound PsyCap Scale (CPC-12, [70]), which is a compound of hope, optimism, self-efficacy and resilience. Participants rated the 12 items on a 6-point scale ranging from 1 = *‘absolutely disagree’* to 6 = *‘absolutely agree’* (e.g. ‘*I can think of many ways to reach my current goals*.’).

#### Data analysis

The data analysis was performed analogous to studies 1 and 2 using R statistical software [47] and SPSS [49].

### Results

The results of the CFA for the SUDCO-G showed an acceptable fit according to Hu and Bentler [27]: *χ²* (246) *=* 586.92 (*p* < .001), CFI = .933, TLI = .925, RMSEA [95% CI] = .079 [.070, .087], SRMR = .052. The result was very similar to the model fit in the original study [14] and to the model fit in study 1 and 2, therefore supporting hypothesis 1 and reapproving the results of study 1 and 2. The factor loadings ranged between .72 and .91, all of them significant on their respective factor at *p <* .01.

Table 5 presents descriptive statistics, bivariate correlations and Cronbach’s *α* and McDonald’s *ω* for the study variables. Cronbach’s *α* and McDonald’s *ω* for all scales were very good. As expected SUB showed statistically significant positive correlations with general strengths use, meaning of work and PsyCap. POSSU was also statistically significantly correlated with general strengths use and meaning of work. Hypotheses 7 through 9 were therefore supported.

**Table 5:** Descriptive statistics and inter-correlations for Study 3 (N = 295).

Variables	*M*	*SD*	*α*	*ω*	1.	2.	3.	4.
1. POSSU	4.46	1.44	.96	.97	1			
2. POSDC	3.48	1.58	.90	.92	.55**	1		
3. SUB	5.22	1.23	.94	.96	.75***	.35***	1	
4. DCB	4.43	1.36	.92	.94	.54***	.61***	.52***	1
5. Age	31.24	10.65	-	-	.11	-.07	.16	-.12*
6. Tenure	51.12	87.33	-	-	.03	-.05	.07	-.07
7. Hours	30.47	15.15	-	-	.19*	.20**	.19**	.09
8. GSU	5.13	0.97	.95	.96	.63***	.33***	.75***	.43***
9. MoW	3.43	0.80	.91	.94	.59***	.35***	.55***	.47***
10. PsyCap	4.44	0.66	.89	.92	.41***	.18**	.55***	.31***

Notes. POSSU = perceived organizational support for strengths use, POSDC = perceived organizational support for deficit correction, SUB = strengths use behavior, DCB = deficit correction behavior, Age = age in years, Tenure = tenure in months, Hours = hours worked per week, GUS = general strengths use, MoW = meaning of work, PsyCap = psychological capital; p-scores:

* < .05,

** < .01,

*** < .001.

### Measurement invariance analysis

In order to examine the comparability of the SUDCO-G with the original version of the SUDCO, we tested for measurement invariance using the South African and Dutch samples from Van Woerkom et al. [14]. We used the ‘*lavaan*’ package [46] of R statistical software [47]. In order to define invariance we followed the recommendations of Cheung and Rensvold [71] using ΔCFI < .01 as criterion.

Table 6 presents the results of the measurement invariance analysis. The comparison of three samples (Dutch, German and South African) supports weak but not strong measurement invariance, allowing only for meaningful comparisons of the relationships with latent variables (loadings) between the different groups (metric invariance; [72]). We also checked the variances of the intercepts in order to test for partial invariance. Deficit correction and support for deficit correction items showed the highest variances. However, freeing the parameters for these two scales did not result in strong measurement invariance.

**Table 6:** Measurement invariance.

Model	χ²(df)	CFI	BIC	RMSEA	Model comp	Δχ²(Δdf)	ΔCFI scaled	Decision
M1: configural	2411.655(738)**	.931	119765.740	.064				
M2: weak	2570.316(778)**	.926	119646.567	.064	M1	162.876(40)**	.0005	Accept
M3: strong	3073.854(818)**	.907	119951.290	.070	M2	571.471(40)**	.019	Reject
M4: strict	3244.031(826)**	.901	120096.136	.073	M3	204.095(8)**	.007	Reject

Notes. *N* = 1669, group 1 *n* = 699, group 2 *n* = 837, group 3 *n* = 133.

### Conclusion

Study 3 supported the findings from study 1 and study 2 concerning the SUDCO-G factor structure. Extending the findings by Van Woerkom et al. [14], the results show that the SUDCO-G shows concurrent validity for its subscale SUB with the German general strengths use scale. Meaning of work and PsyCap are also positively related to SUB and POSSU.

## Discussion

### Limitations

The results of this study should be interpreted with the following limitations in mind.

First, the participants were recruited and participated online. Therefore, the study might only reach certain people and lack generalizability. However, according to Gosling, Vazire, Srivastava, and John [73] online recruitment and participation should only be of marginal effect to the results. They emphasized that samples using online recruitment are as diverse, adjusted, and at least as good in quality as most traditional methods. Also, two of the three samples had a substantially higher percentage of female participants. Generalizability of our results can also be questioned due to the use of nonprobability samples.

Also this study used cross-sectional data and self-ratings. The aim of this study was to validate the German version of the SUDCO-G. The obtained results allow no inferences about causality as the chosen design was a cross-sectional one. The issue of using self-ratings and common method variance is debated in work and organizational psychology. So far it is unclear if there is a common method problem to the results or how big the problem might be [74–77].

### Conclusion

The development of the SUDCO [14] made it possible to investigate its constructs in the English-speaking population. The aim of this study was to adapt and validate the SUDCO for the German-speaking population. In three studies the SUDCO-G was found to be a reliable and valid instrument to measure the four dimensions POSSU, POSDC, SUB and DCB.

In general, the results of the original study by Van Woerkom et al. [14] were similar to the results of this study and all hypotheses are largely supported. Nearly all statistically significant correlations of the original study have also been statistically significant in this study and all other correlations are pointing in the same directions.

Nonetheless, there are some differences that should be pointed out. The two correlations between POSDC and exhaustion and DCB and cynicism, which were both statistically significant negative correlations in the original study, were not statistically significant in this study. This might be due to the different measurement tools we used to assess exhaustion and cynicism in both studies. Another difference can be found when comparing the results for perceived supervisor support. The reported positive correlations in the original study were much lower (*r* = .16 - .23) than in this study (*r* = .32 - .65). This might again be due to the use of a different measurement tool. For all the other constructs the correlations are about the same size, although there is a trend of some correlations being slightly bigger in the original study.

Also POSSU and SUB show statistically significant positive correlations with a general measure of strengths use, supporting convergent validity. Meaning of work and PsyCap show positive correlations with POSSU and SUB and also with POSDC and DCB. Although the positive correlations of POSDC and DCB with those constructs are smaller than those with POSSU and SUB, they are still statistically significant. We would like to point out that the meaning of work relates to both aspects, SUB (*r* = .55) and DCB (*r* = .47) comparably.

Also means between German, South African and Dutch samples cannot be compared as we only found weak measurement invariance. Especially the content of deficit correction seems to have been perceived differently between these countries, as the inspection of the modification indices suggest. This could be due to cultural differences concerning learning from errors. A recent study by Horvath, Klamar, Keith and Frese points out that people deal and learn differently from errors across countries [78]. We would argue, that dealing with and learning from errors is important in correcting your own deficits. Likewise, how this is generally dealt with in a country or society might shape the organization’s support in dealing with deficits. Although their study examines different countries than ours, this might be one possible explanation for the cultural differences we found, that needs further examination.

#### Practical implications

As clearly demonstrated, the four dimensions of the SUDCO are associated with criteria that are beneficial for individuals and organizations. Thus, facilitating the behaviors of strengths use and deficit correction at the workplace and enhancing organizational support for both behaviors seems strongly advisable.

The SUDCO-G might be a helpful instrument for future use in organizations, especially for human resources departments. By assessing its four dimensions in a specific context practitioners can figure out the optimal approach for employee development or performance improvement measures. With this instrument practitioners have a tool to find the optimal mix of support for strengths use and deficit correction to get ideal results in many human resources practices (e.g. individual development plans, employee orientation interviews, team composition, etc.). Organizations in the German-speaking countries could therefore benefit from this instrument. As we only conducted correlational studies, we can draw no conclusion on the causality of the relations of strengths use and deficit correction with the criteria we examined. So for example, enhancing the meaning of work could lead to more strengths use and vice versa. Our current results therefore provide no advice on which approach would be the better lever.

### Future research

Although past research has already revealed the importance of strengths use and deficit correction behavior and organizational support for the well-being of the individual and the organization, most organizations today still fail to implement a strategy to stimulate these important behaviors. Future research should therefore engage in the development and evaluation of interventions to increase POSSU, POSDC, SUB and DCB altogether or one at a time. Organizations need to be made aware of the potentially positive outcomes of engaging with these issues and of the risks of ignoring them.

In order to develop good interventions programs, more research is needed on the interplay between strengths use and deficit correction. Examining if there is an optimal ratio between the two could provide further advice for leaders and HR departments in order to decide on when and how often to address deficit areas and optimally support their employees’ growth.

Furthermore, the standardization of the SUDCO-G needs to be addressed. Contemporary norms are helpful for a meaningful interpretation of the actual scores of the questionnaire. This could help to discover individuals, teams or departments of organizations that for example desperately need improvement in one area of the questionnaire or another. It could also help to discover parts of the organization that are already doing well on the four dimensions and could therefore be utilized as an example.

Further research is also needed on the actual value of SUB and DCB over a wide variety of situations. Practitioners could then be provided with more guiding advice on when to focus on SUB or when to focus on DCB or when a combination of both constructs might be most valuable.

## Supporting information

S1 Fig (TIF)

S1 Dataset (CSV)

S2 Dataset (CSV)

S3 Dataset (CSV)
