# Supplementary figures and images for: Adaptation and validation of a German version of the Strengths Use and Deficit Correction (SUDCO) questionnaire
Source: PLoS One. 2021 Jan 7;16(1):e0245127. doi: 10.1371/journal.pone.0245127 (PMC7790259; doi:10.1371/journal.pone.0245127)

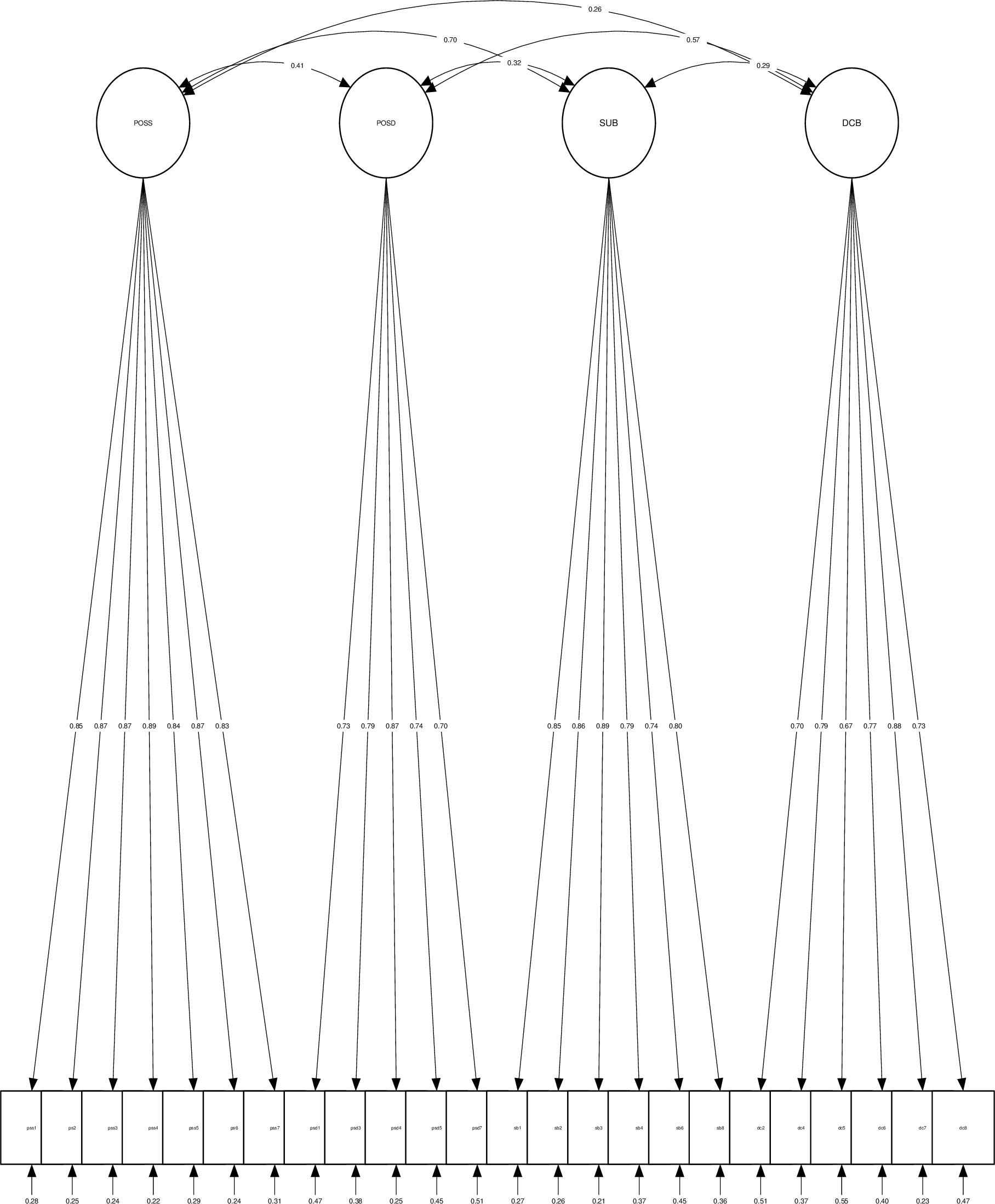

Supplement: S1 Fig — (TIF) [file pone.0245127.s001.tif]
